# Impairments of Social Motor Synchrony Evident in Autism Spectrum Disorder

**DOI:** 10.3389/fpsyg.2016.01323

**Published:** 2016-08-31

**Authors:** Paula Fitzpatrick, Jean A. Frazier, David M. Cochran, Teresa Mitchell, Caitlin Coleman, R. C. Schmidt

**Affiliations:** ^1^Department of Psychology, Assumption College, WorcesterMA, USA; ^2^Department of Psychiatry, University of Massachusetts Medical School, WorcesterMA, USA; ^3^Department of Psychology, College of the Holy Cross, WorcesterMA, USA

**Keywords:** social synchrony, autism spectrum disorders, social dysfunction, social dynamic behavior, coupled oscillators

## Abstract

Social interactions typically involve movements of the body that become synchronized over time and both intentional and spontaneous interactional synchrony have been found to be an essential part of successful human interaction. However, our understanding of the importance of temporal dimensions of social motor synchrony in social dysfunction is limited. Here, we used a pendulum coordination paradigm to assess dynamic, process-oriented measures of social motor synchrony in adolescents with and without autism spectrum disorder (ASD). Our data indicate that adolescents with ASD demonstrate less synchronization in both spontaneous and intentional interpersonal coordination. Coupled oscillator modeling suggests that ASD participants assembled a synchronization dynamic with a weaker coupling strength, which corresponds to a lower sensitivity and decreased attention to the movements of the other person, but do not demonstrate evidence of a delay in information transmission. The implication of these findings for isolating an ASD-specific social synchronization deficit that could serve as an objective, bio-behavioral marker is discussed.

## Introduction

Individuals with autism spectrum disorders (ASD) exhibit numerous impairments in social interaction that typically persist throughout adolescence and adulthood (Ballaban-Gil et al., 1996; Howlin et al., 2004; Billstedt et al., 2005; Eaves and Ho, 2008; American Psychiatric Association [APA], 2013). These deficits impact mental and physical development, learning, and behavioral functioning across settings and are the main reason that even high functioning individuals have difficulty contributing to the workforce in adulthood (Arnett, 2000; Howlin et al., 2004). Past research has found that weaknesses in social competence of individuals with ASD are comprised of deficits in a number of areas including social cognitive (Baron-Cohen, 1995) and social perceptual processes (Klin et al., 2002). These deficits, however, are difficult to treat and there are few evidence-based interventions available to target them. One noteworthy characteristic of social interactions that has not been the focus of much research is the coordination and timing of bodies that occur in jointly created actions. For example, when two people carry on a conversation, they take turns speaking and synchronizing their hand gestures (Wilson and Wilson, 2005; Louwerse et al., 2012) or match each other’s stride length and step in synchrony when walking together (van Ulzen et al., 2008). The temporal nature of such social motor synchronization remains an overlooked dimension of social communication in ASD research.

This is unfortunate because there is a large body of research that suggests that how we move our body or express ourselves via our body “language” has a substantial impact not only on how others perceive us, but also on our own mental states and physiological well-being. For example, synchronizing one’s body with another person has been found to be vital for maintaining critical aspects of successful social interaction including interpersonal responsiveness, social rapport and other-directedness (Bernieri et al., 1994; Lakin and Chartrand, 2003), positive self-other relations (Miles et al., 2009; Seger and Smith, 2009), cooperation (Wiltermuth and Heath, 2009; Valdesolo et al., 2010; Reddish et al., 2013, 2014), and verbal communication and comprehension (Semin, 2007; Shockley et al., 2009).

Consequently, this interpersonal synchrony can be thought to reflect psychological connectedness and research with adults has found that interpersonal synchrony breaks down in social pathology. For example, breakdowns in synchronization are associated with marital dissatisfaction (Julien et al., 2000), as well as psychological disorders such as schizophrenia (Ramseyer and Tschacher, 2011; Varlet et al., 2012) and borderline personality disorder (Gratier and Apter-Danon, 2009). The psychological importance of social synchronization is also underscored by research that found that manipulating an individual’s body into different poses has the ability to change their perception, emotions, and even impact physiological changes within an individual (Strack et al., 1988; Carney et al., 2010). That is, the way we move our body influences our own mental and physiological states, the social judgments others make of us and can consequently foster or inhibit the social connection we have with others.

In its broadest sense, interpersonal synchronization can be defined as “a range of social communication activities and constructs including joint attention, imitation, turn-taking, non-verbal social communicative exchanges, affect sharing and engagement” (Charman, 2011). Such social communication requires synchronization in both time and content (Kinsbourne and Helt, 2011; Delaherche et al., 2012). Given that impairments in social interaction and communication are core features of ASD (American Psychiatric Association [APA], 2013), the role of various aspects of social synchronization, broadly defined, has been the focus of much research.

For example, imitation has been widely studied because imitation is thought to be a precursor to more complex social cognition such as joint attention and understanding agency (Meltzoff, 1990, 2009). A number of researchers have found that imitation is disrupted in ASD and have proposed that an atypically functioning mirror neuron system may be the underlying mechanism (Rogers and Pennington, 1991; Charman et al., 1997; Williams et al., 2001; Rogers et al., 2003; Gallese, 2006; Oberman and Ramachandran, 2007; Colombi et al., 2009; Rizzolatti and Fabbri-Destro, 2010). Other research, however, suggests that some children with ASD do not have deficits in imitative movements and that the mirror neuron system of the social brain may not be damaged (Hamilton et al., 2007; Gowen et al., 2008; Fan et al., 2010). Similarly, Kinsbourne and Helt (2011) argue that individuals with ASD are capable of imitation but just produce it less frequently, especially in more naturalistic situations. They suggest this is due not to imitation problems and a damaged mirror neuron system but rather caused by a lack of social attention. Moreover, Gernsbacher et al. (2008) and Dowd et al. (2010) have suggested alternate processes, namely, motor control problems, as potentially important for understanding social interaction.

Much social synchronization research has been concerned with coding of whether general activity or certain behavioral contents are synchronized (i.e., similar gestures are occurring together or at a lag). Condon was one of the early researchers to code for the timing of general activity and he proposed that synchronized bodily coordination was disturbed in social pathologies generally and in particular in children with ASD (Condon, 1982). Similarly, Trevarthen and Daniel (2005) coded for synchronous emotional arousal, initiation, and changing of attention and reported a lack of reciprocity in the parent–child exchanges of an infant with ASD and Feldman’s (2007) research, based on coding of mutual gaze, shared attention, and arousal, has found that synchronization is predictive of social outcomes such as attachment and empathy. Furthermore, Oberman et al. (2009) found that children with ASD differed in latency to produce facial mimicry, but not in the amount they mimicked, suggesting problems in interpersonal synchrony may be due to disruptions in timing. A breakdown of the temporal synchronization of specific kinds of speech behaviors have also been reported in adolescents with ASD. For example, Feldstein et al. (1982) found that the ability of adolescents with autism to synchronize the timing of their speech to that of a conversational partner was poor and de Marchena and Eigsti (2010) discovered that adolescents with ASD do not synchronize gestures with speech.

The research reviewed above relies largely on a behavioral coding of specific gestures (content) to evaluate social synchronization. Such behavioral coding methods rely on identifying discrete segments of behavior and analyzing the sequencing or timing between them but are time consuming to perform and rely on highly skilled coders. Moreover, such behavioral coding is not particularly well-suited for understanding the full temporal patterning of social synchronization in that it is discrete and not fine-grained enough to capture the complex, time-dependent dynamic organization of interpersonal synchrony. Consequently, a methodology that investigates the “process” of the social activity generally (rather than specific behaviors) in order to ascertain the time unfolding nature of social interaction may provide measures with more resolution that might deepen our understanding of the social synchronization in general and its deficits in ASD specifically.

A coordination dynamics approach to behavior (Kelso, 1995) provides a framework for the development of such a research methodology. This approach involves recording continuous time-varying process measures of behavior as they unfold and then analyzes the dynamical structure of behavior using time-series analysis techniques. These techniques allow for a more discerning measurement of behavioral coordination by evaluating the synchronization (patterning and strength) of system components as they change over time (Haken et al., 1985; Strogatz, 2003). The temporal resolution of this approach allows for the capture of subtle dimensions of coordination that are typically missed by gross outcome measures. The ability to index subtle changes in the patterning and stability of coordination will allow us to determine whether such differences are related to the variations in social competence that are observed in adolescents with ASD.

This coordination dynamics approach has been used to model social coordination (Schmidt and Richardson, 2008; Schmidt et al., 2011). For example, both intentional coordination (directed by an explicit social goal of the people interacting) as well as spontaneous coordination (outside of the awareness of the two people interacting) of the movements of two people interacting have been modeled using a coupled oscillator dynamic for both simple laboratory tasks (Schmidt et al., 1998, 2011; Richardson et al., 2005) as well as more naturalistic interactions (Schmidt et al., 2012, 2014). In the dynamical modeling of this interpersonal synchronization, individual limbs of the two people are treated as embodying oscillators that are linked via perceptual coupling (Richardson et al., 2005; Schmidt et al., 2011, 2012; Schmidt, 1988, unpublished).

A task that has been used to study the both intentional and spontaneous interpersonal coordination is a methodology in which two people coordinate handheld pendulums swung from the wrist joint in the sagittal plane (using radial-ulnar abduction–adduction). This methodology has demonstrated that the strength of interpersonal synchronization is dependent upon many different physical as well as psychological variables (see Schmidt and Richardson, 2008 for a review) and can be understood in terms of a dynamical model of synchronization.

A synchronization dynamic model (Varlet et al., 2012) used to understand pendulum coordination utilizes a non-linear coupling of two limit-cycle oscillators:

(1) $$\begin{array}{c} {\ddot{x}}_{1}+\delta {\dot{x}}_{1}+\lambda {\dot{x}}_{1}^{3}+\gamma x_{1}^{2}{\dot{x}}_{1}+\omega^{2}x_{1}=K_{1}({\dot{x}}_{1}-{\dot{x}}_{2\tau_{1}})[a+b{\left(x_{1}-x_{2\tau_{1}}\right)}^{2}]\;\\ {\ddot{x}}_{2}+\delta {\dot{x}}_{2}+\lambda {\dot{x}}_{2}^{3}+\gamma x_{2}^{2}{\dot{x}}_{2}+\omega^{2}x_{2}=K_{2}({\dot{x}}_{2}-{\dot{x}}_{1\tau_{2}})[a+b{\left(x_{2}-x_{1\tau_{2}}\right)}^{2}] \end{array}$$

where *x*_1_ and *x*_2_ represent the positions of the two oscillators and the dot notation represents derivative with respect to time. The left side of the equations represents the limit cycle dynamics of each oscillator determined by a linear stiffness parameter (ω^2^) and damping parameters (δ, λ, γ) and the right side represents the coupling function determined by strength parameters *a* and *b*. This model predicts that even if the two pendulums have different (inherent) eigenfrequencies (which can be induced by manipulating the length or mass of the pendulum) and two people are asked to coordinate the movements of two pendulums, they are able to do so and achieve a common tempo. However, the person swinging the pendulum that prefers to move more slowly (e.g., the one with the lower eigenfrequency) lags *slightly* behind the person swinging the pendulum that prefers to move faster. The magnitude of this lagging and leading (phase shift) is determined by the interplay of the difference between the eigenfrequencies of the pendulums (the degree of frequency detuning) and two model parameters—one that indexes the coupling strength of the two oscillators (*K*_1_ and *K*_2_ corresponding to the coupling strengths of the oscillators 1 and 2) and another that indexes the rate (delay/advance) of information transmission (x_2τ_1__ and ẋ_2τ_1__ corresponding to the position and the velocity of the oscillator 2 at a previous time point *t*-τ_1_ and the parameters x_1τ_2__ and x_1τ_2__ corresponding to the position and the velocity of the oscillator 1 at a previous time point *t*-τ_2._

Using such a dynamical model to understand how synchrony breaks down in social deficits has the distinct advantage of allowing one to infer which dynamical components of the model are underlying the impairment. Varlet et al. (2012) adopted this strategy and found that individuals with schizophrenia exhibited both a lower coupling strength and an information transmission delay when performing intentional interpersonal coordination. However, they did not find any disruptions in spontaneous interpersonal coordination. These findings suggest that individuals with schizophrenia may not be attending to others or may be delayed in their responses during social interactions when they are interacting with them under an explicit social goal to coordinate. Del-Monte et al. (2013) have extended this work and found that first-degree relatives of patients to schizophrenia demonstrate the same overall pattern of synchronization impairments as individuals with schizophrenia. Namely, the first-degree relative pairs also demonstrated larger phase lag and greater variability but only for the intentional rhythmic coordination of pendulums. The results of these two studies suggest that social motor synchronization may be part of schizophrenia’s core deficits and may provide a bio-behavioral marker for the disorder.

Relatedly, to demonstrate the feasibility of using dynamical measures of social synchronization to investigate the social deficit in those with ASD, Fitzpatrick et al. (2013a,b) designed a battery of movement tasks to investigate the dynamics of social synchrony in children (6–10 years old) with ASD. They also utilized traditional cognitive measures of social competence (joint attention, theory of mind, intentionality, and cooperation) and several social motor measures including imitation, synchronization and an interpersonal hand-clapping game. Findings yielded significant relationships between social cognitive and social synchrony measures and a principal components analysis revealed three different factors (social attention, social knowledge, and social action) as important for characterizing embodied social competence. These findings suggested that social competence is a complex construct and identified social synchrony as a potentially important pathway for understanding the social problems of children with ASD.

Taken together, the research discussed above suggests that social synchronization is a potentially important pathway for understanding the social problems characteristic of people with social deficits. The current study extends the previous work by employing a pendulum coordination task to examine the content and timing of social motor synchronization of adolescents with ASD. The aim of this study is to determine whether adolescents with ASD exhibit an interpersonal synchrony deficit and whether this can be used to differentiate adolescents with and without ASD. In particular, we are evaluating (a) whether disruptions are evident in both intentional and spontaneous coordination; and (b) which components of the coupled oscillator dynamic are impaired (e.g., coupling strength only, information transmission only, neither or both). An impairment in coupling strength would reveal difficulty in attending to social cues, an impairment in information transmission would suggest problems with detecting and processing the information in time for an appropriate response, and disruptions in both would indicate problems with both attending to social cues as well as processing the information. The use of a social motor synchronization task allows for a precise, objective, and dynamical measure of synchronization and a more nuanced exploration of the temporal nature of synchronization. In addition, the *direct* dynamical modeling available using the pendulum paradigm will allow us to explore whether a social synchronization deficit is general or specific to a disorder (i.e., different for schizophrenia and ASD).

## Materials and Methods

### Participants

A total of 18 adolescents paired with one of their parents participated in this study. There were nine adolescents with a diagnosis of ASD (eight males, one female, average age 13.67 ± SD years, range 12–17) and nine control adolescents (seven males, two females, average age 14.44 ± SD years, range 12–16). There was one adolescent with ASD who was left-handed; all other participants in both groups were right-handed.

The participants with ASD had previously been diagnosed by a licensed clinical psychologist or psychiatrist based on Diagnostic and Statistical Manual of Mental Disorders, 4th Edition, Text Revision (DSM-IV-TR) criteria (American Psychiatric Association [APA], 2000) and diagnosis was confirmed using the Autism Diagnostic Observation Schedule, 2nd Edition (ADOS-2) (Lord et al., 2012). The ADOS-2 is a semi-structured, standardized assessment of communication, social interaction, and play for individuals referred because of a question of a possible diagnosis of autism. Control participants also completed the ADOS-2. Five participants were administered Module 3 whereas 13 were administered Module 4 based on their developmental and language level. The mean ADOS scores for the two groups were significantly different from each other and confirmed group membership (**Table 1**). The groups were matched for chronological age and the Wechsler Abbreviated Scale of Intelligence (WASI) IQ score (Wechsler, 2011) for both groups was in the normal range of 85–115, although the WASI IQ score of the ASD group was slightly lower than the control group (**Table 1**).

**Table 1:** Participant characteristics and clinical phenotyping.

	ASD (*n* = 9)		Neurotypical (*n* = 9)		Group difference	
	Mean	SD	Mean	SD	*t*(16)	*p*
Chronological age (years)	13.67	1.94	14.44	1.13	-1.04	0.31
WASI vocabulary	52	10.99	63.78	6.72	-2.74	0.01
WASI matrix	49.78	7.12	55.44	8.75	-1.51	0.15
WASI IQ	101.78	13.84	117.22	13.15	-2.43	0.03
**ADOS**					
Communication	3.11	0.93	0.22	0.44	8.44	<0.001
Social interaction	5.44	2.19	0.11	0.33	7.24	<0.001
Communication and social interaction total	8.56	2.92	0.33	0.5	8.33	<0.001
Stereotyped behaviors and restricted interests	2.0	1.41	0	0	4.26	0.001

All parents of participants gave informed, written consent for their children to take part in the study, and adolescents also provided assent to participate. The project was approved by the University of Massachusetts Medical School (Docket # H00001602) and Assumption College Institutional Review Boards (IRB # 2012-17, March 18, 2013).

Participants were recruited from local communities through print advertising, a recruitment brochure, email, social media, and community events. Recruitment material was posted on various community and University of Massachusetts Medical School websites.

### Apparatus

**FIGURE 1:**
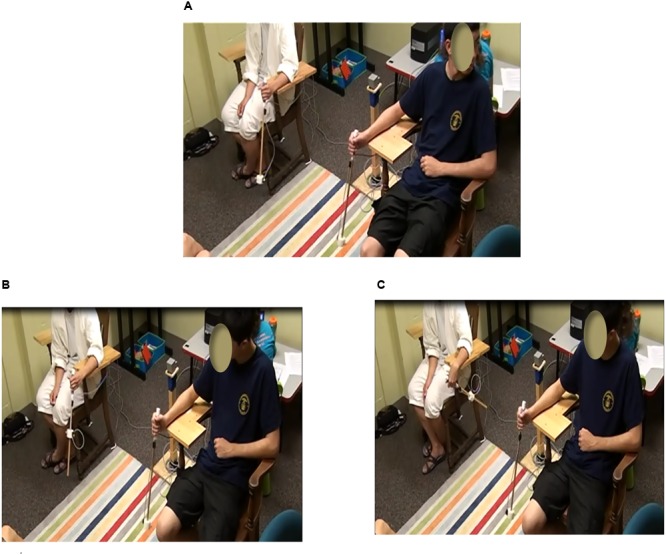
**Experimental set-up for the spontaneous and intentional pendulum task.** Participants sat on chairs side-by-side while oscillating pendulums. In the spontaneous coordination conditions, participants coordinated their pendulums at their own tempo and looked away from each other’s pendulum during the “not looking” conditions **(A)** and toward the partner’s pendulum during the “looking” conditions. In the intentional coordination condition, participants looked at each other’s pendulum and swung the pendulums in either an in-phase coordination pattern **(B)** or an anti-phase coordination pattern **(C)**.

Participants sat on chairs 1 m apart, facing the same direction (**Figure 1**). Each chair had a forearm support attached to the inside of the chair parallel to the ground. This ensured that the handheld pendulums would be swung about the wrist in the sagittal plane and participants would have an unobstructed view of their partner’s pendulum. Adolescents swung the pendulums with their dominant hand and parents swung the pendulums with the non-dominant hand.

The time-series motions of the pendulums were recorded at 100 Hz using a magnetic motion tracking system (Polhemus Liberty, Polhemus Corporation, Colchester, VT, USA) and 6-D Research System software (Skill Technologies, Inc., Phoenix, AZ, USA). A sensor was attached to the end of each pendulum to record the angular displacement of the pendulum. The time series of participants were low-pass filtered using a 10 Hz Butterworth filter.

### Pendulum Preferred Frequency of Oscillation Manipulation

Two handheld pendulums, each composed of a wooden dowel that was 54 cm in length and had a 100 g weight attached at either the bottom or the middle of the pendulum, were used. The placement of the weight manipulated the inertial loading of the pendulum, and hence, the preferred frequency of oscillation. Pendulums weighted at the middle have a lower inertial load and a higher preferred frequency of oscillation whereas pendulums weighted at the bottom have a larger inertial load and a lower preferred frequency of oscillation (Schmidt and Turvey, 1994; Schmidt and O’Brien, 1997). The pairing of the two pendulums resulted in three pendulum combination conditions for participant pairs that reflect differential inertial loadings of the pendulums and differential preferred frequencies of oscillation: 0 (no inertial difference between pendulum conditions, both adolescent and parent have pendulum weighted at bottom, no preferred frequency of oscillation difference); 1 [parent had pendulum with higher inertial loading (mass at bottom) and adolescent had pendulum with lower inertial loading (mass at middle), adolescent had higher preferred frequency of oscillation and should lead the coordination]; and -1 [adolescent had pendulum with higher inertial loading (mass at bottom) and parent had pendulum with lower inertial loading (mass at middle), parent had higher preferred frequency of oscillation and should lead the coordination].

### Social Synchronization Tasks

To measure social synchronization, adolescent–parent pairs swung three combinations of pendulums as described above and the movement time series of the adolescent’s and parent’s pendulums were recorded using the Polhemus movement capture system. Two different synchronization tasks were performed, spontaneous synchrony and intentional synchrony.

#### Spontaneous Social Synchronization

To evaluate spontaneous synchrony, 90 s trials were completed in which each participant swung his/her pendulum at a comfortable tempo and maintained that tempo. During the control trial segments (the first and last 30 s) participants were looking away from their partner’s pendulum and during the spontaneous coordination experimental segment (middle 30 s) the participants were looking at each other’s pendulums (**Figure 1A**). Trials, including the not looking (control) segments and looking (spontaneous coordination) segments, were completed for each of the three pendulum combination conditions. Two replications per pendulum condition were completed for a total of six spontaneous synchrony trials.

#### Intentional Social Synchronization

To evaluate intentional synchrony, participant pairs were instructed to coordinate their pendulum swinging with their partner in either an in-phase pattern so their pendulums were in the same portion of their cycles at the same time (**Figure 1B**) or anti-phase pattern so that their pendulums were in opposite portions of their cycles at the same time (**Figure 1C**). Trials were 60 s, with two replications for each pendulum combination condition, for both in-phase and anti-phase coordination resulting in a total of 12 intentional coordination trials (6 in-phase, 6 anti-phase).

### Social Synchronization Measures

Relative phasing of the adolescent’s and parent’s pendulum movements was used to evaluate the degree and pattern of rhythmic synchronization. Relative phase is an angle that measures where one rhythm is in its cycle (i.e., its phase) with respect to where another rhythm is in its cycle. If two rhythms are in identical parts of their cycles, they have a relative phase of 0° and are in-phase. If two rhythms are in opposite parts of their cycles, they have a relative phase of 180° and are in anti-phase. A continuous relative phase time series was computed from the two angular positions of pendulums using the Hilbert transform (Pikovsky et al., 2003).

#### Spontaneous Social Synchronization Task

The degree of synchronization was evaluated by a measure of relative phase variability. We computed the circular variance (Batschelet, 1981) of the relative phasing between the two participant’s movements from the continuous relative phase time series. This measure yields an index of synchronization between 0 and 1 with 1 reflecting a perfect synchronization and 0 reflecting an absence of synchronization. The circular variance represents the proportion of relative phases relationships visited by the two time series. A circular variance of 0 means that the two time series never visited the same relative phase relationship more than once. Higher values of circular variance indicate that the two time series repeatedly visited a set of relative phase relationships throughout the trial.

#### Intentional Social Synchronization Task

To evaluate the synchronization that occurred in both intentional in-phase and anti-phase synchronization tasks, two dependent measures were calculated from the relative phase time series. First, circular variance was calculated to measure the overall degree of synchronization. As mentioned above, a circular variance of 0 indicates no synchronization and a circular variance of 1 indicates perfect synchronization.

Second, mean circular relative phase angle (Batschelet, 1981) was calculated from the continuous relative phase time series to determine the phase shift (lag–lead relationship) associated with each dyad’s coordinated rhythmic movements. Positive relative phase angles (phase shifts from intended phase 0° or 180°) indicated that child led the coordination and negative relative phase angles (phase shifts) indicated that child followed the movements of the parent.

### Design and Procedure

Participants completed two separate experimental sessions, approximately 1 week apart. In the first experimental session at the University of Massachusetts Medical School, clinical phenotyping was completed including the ADOS-2 and the WASI Matrix Reasoning and vocabulary subtests and lasted approximately 3 hours. Additional clinical phenotyping measures were administered as part of a larger study during this session but are not reported here.

In the second visit, the social synchronization tasks were completed. All participant pairs completed the spontaneous synchrony trials at the start of the experimental session to prevent experimental task demands from influencing performance. The order of presentation of the in-phase and anti-phase intentional synchrony trials was counterbalanced across participants—half of the participant pairs completed in-phase trials followed by anti-phase trials and half completed anti-phase trials followed by in-phase trials. Two additional experimental tasks were also completed as part of a larger study but they are not being reported here.

To summarize the design of the experiment, diagnosis group (ASD, neurotypical control) was a between-subject variable. Group differences in clinical phenotyping were evaluated with independent samples *t*-tests. In the spontaneous social synchrony task, diagnosis group was a between-subject variable, and pendulum combination condition [-1 (adolescent with higher loading, parent should lead), 0 (no differential loading), 1 (parent with higher loading, child should lead)], and looking condition (1st 30 s, not looking; 2nd 30 s looking; 3rd 30 s not looking) were within-subject variables. The circular variance of relative phase for the spontaneous synchrony task was analyzed with a 2 (diagnosis group) × 3 (pendulum combination) × 3 (looking) analysis of variance (ANOVA). For the intentional synchrony task, a 2 (diagnosis group) × 3 (pendulum combination) × 2 (phase mode, in-phase or anti-phase) ANOVA was used to analyze the dependent measure, circular variance of relative phase. Circular variance values were standardized using a Fisher’s z-transformation before the statistical analyses were performed. Bonferroni *post hoc* tests were used as necessary to determine the nature of the effects. To determine whether IQ affected the results, all the ANOVAs reported below were run with IQ as a covariate. IQ was not a significant factor in any of the analyses, nor did IQ occur as a variable in any significant interactions. Therefore, results are reported below without IQ as a covariate.

## Results

### Was There an ASD Synchrony Deficit for Spontaneous Coordination?

**FIGURE 2:**
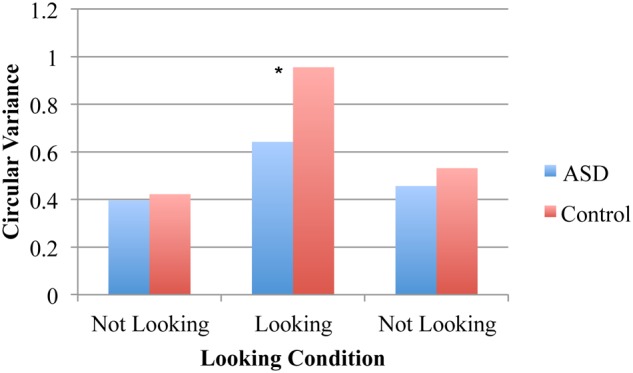
**Circular variance for spontaneous coordination conditions.** No entrainment occurred during the two “not looking” conditions but spontaneous phase entrainment was evident in all pairs during the looking condition (i.e., when participants looked at the partner’s pendulum). Overall less phase entrainment was displayed during the looking condition in the ASD pairs than the control pairs (^∗^*p* = 0.03).

For the spontaneous coordination task, an ANOVA on the circular variance of relative phase resulted in a significant main effects of pendulum combination [*F*(2,32) = 9.94, *p* < 0.001, η^2^ = 0.38], looking condition [*F*(2,32) = 23.15, *p* < 0.001, η^2^ = 0.59], and diagnosis group [*F*(1,16) = 5.77, *p* = 0.03, η^2^ = 0.27]. These results indicate that both groups had higher spontaneous entrainment when the pendulums were the same rather than different, that both the groups demonstrated spontaneous entrainment during the looking condition (as evidenced by higher circular variance) and that ASD pairs had less spontaneous entrainment than the control pairs across all trial segments. The latter two main effects were qualified by a significant looking segment and diagnosis interaction [*F*(2,32) = 3.25, *p* = 0.05, η^2^ = 0.17], indicating that there was only a significant group difference for the looking trial segment (*p* = 0.03, η^2^ = 0.25) but not for either of the non-looking segments (both *p* > 0.05, η^2^ = 0.01 and 0.10; **Figure 2**). The interaction between looking condition and pendulum combination was also significant [*F*(4,64) = 3.18, *p* = 0.02, η^2^ = 0.17] suggesting that the degree of synchronization observed depended upon the pendulum combination more for the looking than the non-looking conditions. Neither the interaction between pendulum combination and diagnosis nor the three-way interaction were significant.

### Was There a ASD Synchrony Deficit for Intentional Coordination?

#### Circular Variance

For the intentional synchrony trials, an ANOVA on circular variance of relative phase verified several dynamical model predictions that have been observed before in a number of studies (see Schmidt and Richardson, 2008 for a review). A main effect of phase mode [*F*(1,16) = 157.60, *p* < 0.001, η^2^ = 0.91], revealed that in-phase coordination demonstrated more stable entrainment (0.88) than anti-phase (0.73). A main effect of pendulum combination [*F*(2,32) = 10.63, *p* = 0.001, η^2^ = 0.40] indicated that pendulum combinations with similar pendulums had more stable entrainment than combinations with different pendulums. The phase mode by pendulum combination interaction was not significant [*F*(2,32) = 1.86, *p* = 0.17, η^2^ = 0.10], indicating the influence of pendulum combination was the same for both in-phase and anti-phase coordination.

**FIGURE 3:**
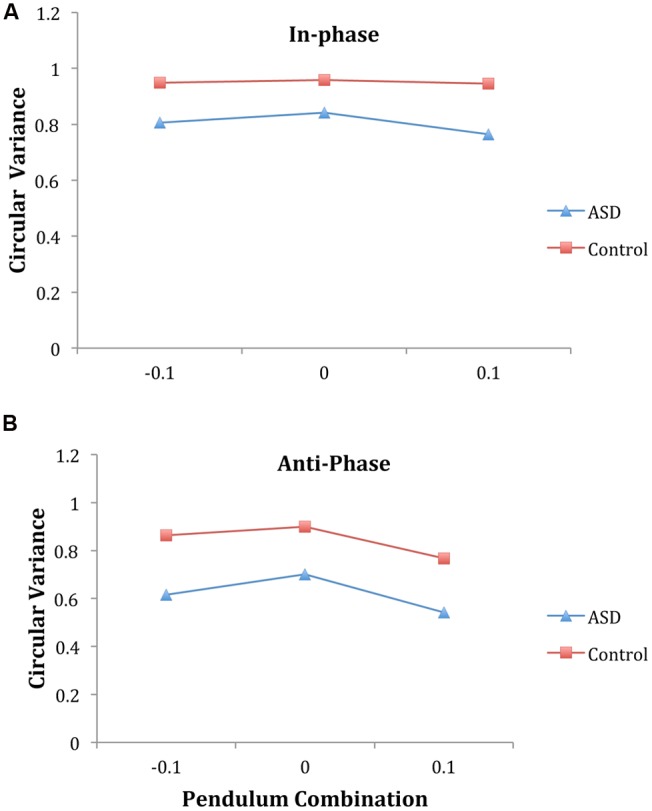
**Intentional coordination phase entrainment (as indexed by circular variance of relative phase) supports the dynamical model predictions.** Namely, entrainment is lower for anti-phase **(B)** than in-phase **(A)** and entrainment is higher for similar pendulum combinations than different combinations. Importantly, the ASD pairs had lower overall entrainment than the control pairs in all conditions. (Note: Convention comparable with Varlet et al., 2012; -0.1 indicates parent should lead, +0.1 indicates child should lead.)

Importantly, across all conditions, a main effect of diagnosis group revealed that ASD pairs had less stable entrainment than control pairs [0.71 and 0.90, respectively, *F*(1,16) = 24.55, *p* < 0.001, η^2^ = 0.61]. The interaction between phase mode and diagnosis was not significant indicating that the ASD group had lower circular variance than the control for both in-phase and anti-phase coordination (**Figure 3**). In addition, the interaction between pendulum combination and diagnosis was not significant, nor was the three-way interaction, suggesting that the influence of pendulum combination was similar for both groups (**Figure 3**), with the ASD group demonstrating an overall lower level of synchronization.

#### Phase Shift

**FIGURE 4:**
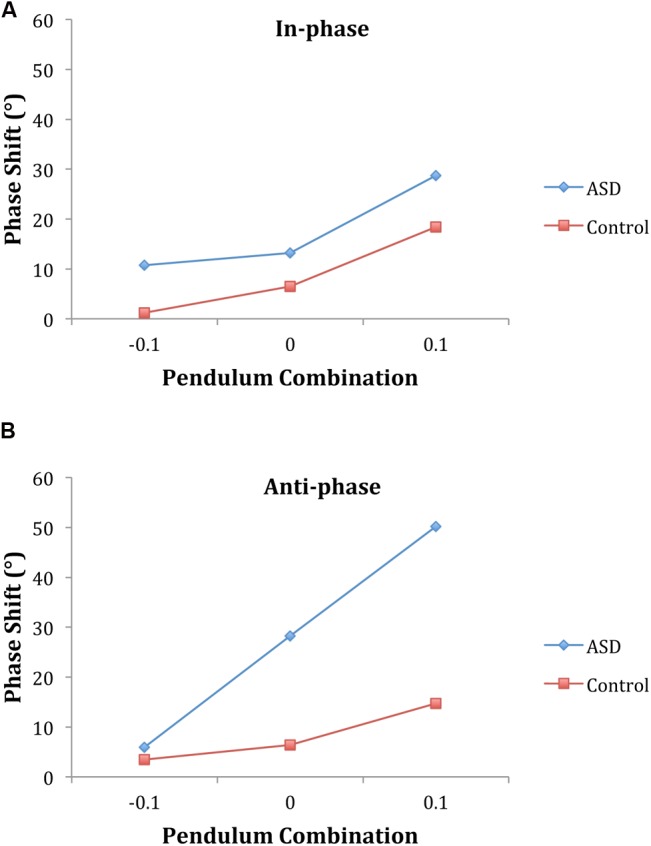
**Phase shift supports the dynamical model predictions for intentional coordination.** The adolescent led less when the parent had the larger pendulum and led the most when they had the larger pendulum. This was true for both groups and was evident for both in-phase **(A)** and anti-phase **(B)** coordination. The phase shift was greater overall for the group with ASD than the controls and the difference between groups was more pronounced for anti-phase than in-phase. (Note: Convention comparable with Varlet et al., 2012; -0.1 indicates parent should lead, +0.1 indicates child should lead.)

The ANOVA on the phase shift (mean relative phase angle) revealed the model-based predicted main effect of pendulum combination [*F*(2,32) = 20.21, *p* < 0.001, η^2^ = 0.56] indicating greater lagging for the person with the larger pendulum. The positive sign of the phase shift values indicate that across both groups the adolescent always led the parent and, there was a trend toward the adolescent with ASD to lead by more [22.82° vs. 8.42°; *F*(1,16) = 3.5, *p* = 0.08, η^2^ = 0.18]. The three-way interaction between pendulum combination, phase mode, and diagnosis was significant [*F*(2,32) = 4.96, *p* < 0.01, η^2^ = 0.24]. Follow-up analyses revealed no group differences for in-phase coordination but an interaction between pendulum combination and group [*F*(2,32) = 5.70, *p* < 0.01, η^2^ = 0.26] for anti-phase coordination suggesting a steeper linear increase in the phase shift with pendulum combination for the ASD group (**Figure 4**).

**FIGURE 5:**
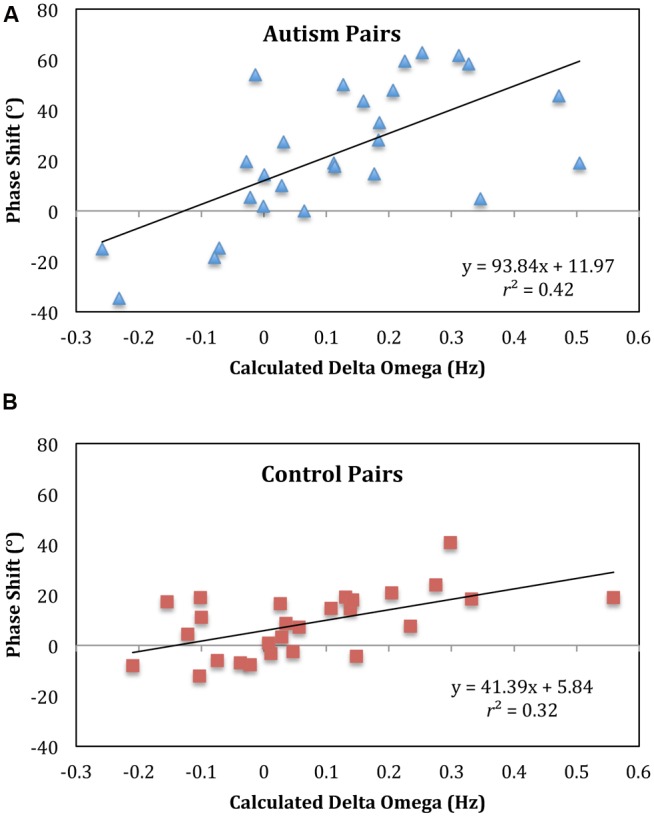
**Phase shift regressions indicate weaker coupling for autism pairs.** A regression with relative phase angle (phase shit) as the dependent variable and calculated delta omega as the independent variable revealed that for both the pairs with autism **(A)** and controls **(B)** with autism and controls the slope and intercept were significantly different from 0. The intercepts were not significantly different between groups but the slopes were. The pairs with autism have a steeper slope, indicative of weaker interpersonal coupling.

To verify this conclusion, a regression analysis was conducted with subject pair mean phase shift values as the dependent variable and actual eigenfrequency differences (frequency detuning value as determined from the unintentional non-looking segments) as the independent variable. As seen in **Figure 5**, there was a significant correlation for the ASD pairs, *r*^2^ = 0.41 (*p* < 0.001), and both the slope and intercept were significantly different from 0 (93.84, *p* < 0.001 and 11.97, *p* = 0.02, respectively). For the control pairs, there was a significant correlation as well, *r*^2^ = 0.32 (*p* = 0.008), and both the slope and intercept were significantly different from 0 (39.75, *p* = 0.008 and 7.47, *p* = 0.008, respectively). The significant slopes in these analyses indicate the model predicted change in phase shift with the eigenfrequency differences of the pendulum pairs whereas the significant intercepts indicate that for both groups the child led the parent in the coordination. A Wald chi-square test showed that the intercepts were not significantly different between groups (*p* = 0.4) but the slopes were significantly different (*p* < 0.001). This slope difference verifies a steeper linear increase in the phase shift with pendulum combination for the ASD group, indicative of weaker coupling. The lack of difference between the intercepts indicates that overall the ASD group did not lead the parent more: there was not an overall phase advance by the ASD group, which would translate into an overall tendency to anticipate.

## Discussion

The findings reported here indicate that adolescents with ASD demonstrated a disruption of both spontaneous synchronization and intentional synchronization. Analysis of circular variance of relative phase confirmed spontaneous social entrainment occurred in both groups, corroborating past research on the ubiquity of spontaneous entrainment. However, the ASD group had weaker spontaneous synchronization during the important second trial segment when participants were viewing each other’s pendulum. For intentional social coordination, the circular variance of relative phase confirmed a number of dynamical model predictions. Namely, for both groups, anti-phase synchronization was weaker than in-phase synchronization and coordinating different pendulums was less stable than coordinating similar pendulums. Importantly though, these analyses also indicate that intentional social synchronization was weaker for the ASD pairs. Thus, our findings on the degree of synchronization using circular variance indicate that ASD participants synchronized less well under conditions in which synchronization occurs spontaneously in the presence of perceptual information of the social partner and in situations when there is an explicit social goal to coordinate with another person (e.g., intentional synchronization).

Evaluation of the pattern of synchronization using the phase shift for the intentional task replicated past findings of frequency detuning in which there was greater lagging for the person with the larger pendulum. Whereas this was true for both groups, the rate of change of this lagging across pendulum pairs was not equal for the two groups: For anti-phase coordination, the ASD pairs showed a steeper lagging slope (**Figure 5**), which indicates, as suggested by the circular variance, that the ASD pairs had weaker synchronization.

In terms of the dynamical model in Eq. 1, these analyses suggest that the ASD pairs assembled a synchronization dynamic in both spontaneous and intentional social coordination situations that has weaker coupling strengths, *K*, than the synchronization dynamic assembled by the control pairs. Such a weakness in dynamical entrainment corresponds to a lower sensitivity and attention to the movements of the other person. Kinsbourne and Helt (2011) have suggested that interpersonal synchrony problems in ASD may be due to a lack of social attention and these findings are consistent with such a claim. That is, given the social nature of the task, the adolescent with ASD was unable to sustain his/her attention to the movement of the partner’s pendulum throughout the trial and hence the synchronization of his/her movements with the partner was lower. Similarly, Bebko et al. (2006) suggest those with ASD may have disruptions in perceiving the temporal aspects of social interactions. This interpretation is reinforced by Koehne et al.’s (2016) findings that adults with ASD did not have synchronization problems when they were asked to synchronize their movements with a dot on a screen. Those participants were told that the movements of the dot were either controlled by a human or a computer, but no social information was present during the task. Participants were not required to use social attention or perception and their synchronization ability was not impaired. Additional research is needed to carefully evaluate the role of animacy on synchronization ability by systematically varying the level of task sociality.

At the same time, whereas there was a slope difference in the regression analysis of frequency detuning (**Figure 5**), there was no intercept difference between the two groups. This lack of a difference suggests that the ASD group did not lead the parent more than the control group and also indicates that there was no group difference in the rate (delay/advance) of information transmission terms in Eq. 1 (Varlet et al., 2012). These findings would indicate that the synchronization problems of adolescents with ASD was due to problems with attention or perception but not with the timing of information transmission. One could also argue that the synchronization difficulties evident in ASD may be the result of motor control problems, which are also common in ASD (Ghaziuddin and Butler, 1998; Pan et al., 2009; Fournier et al., 2010). A number of researchers suggest that motor problems may contribute to the social difficulties of those with ASD (Gernsbacher et al., 2008; Dowd et al., 2010; Bhat et al., 2011). Disentangling the roles that motor control and social attention and perception play in synchronization is needed in future research.

The finding that the majority of children led the parent in synchronization, in both groups, is somewhat surprising. One might have expected adolescents with ASD to be less likely to lead in the coordination. In fact, Warlaumont et al. (2010) found that infants between 16 and 48 months with ASD were more likely to lag in parent–child vocal communicative exchanges while infants without ASD were more likely to lead. Similarly, Varlet et al. (2012) found that individuals with schizophrenia were less likely to lead their partner when performing a social motor synchronization task. The finding that this is not the case in ASD could be suggestive of a lack of attention to the social partner and a lack of reciprocity—the adolescent with ASD is moving the pendulum and the parent is adjusting his/her movements to match the adolescent. This is consistent with the original conception of ASD by Kanner (1943) and Asperger (1944/1991) as a tendency to focus attention inward on their own bodily states even when engaged in tasks that require interaction with the environment.

This specific pattern of disruptions in synchronization ability may be unique to ASD. Whereas participants with schizophrenia have been found to have a social synchrony deficit during intentional synchronization but not spontaneous synchronization (Varlet et al., 2012), participants with ASD demonstrate a less stable entrainment for *both* intentional as well as spontaneous social synchrony. It appears that in individuals with schizophrenia synchronization is disrupted only when there is an explicit social goal, while in ASD the reduction in coupling strength is evident both when there is an explicit social goal to coordinate and when there is no explicit social goal to coordinate. Furthermore, during intentional coordination participants with schizophrenia not only had a weaker coupling strength but also demonstrated a delay in information transmission (Varlet et al., 2012). In contrast, the participants with ASD did not have a deficit in the rate of information transfer. These findings suggest that social synchronization deficit evident in ASD is different from schizophrenia and may be different from other disorders characterized by problems with social interactions. Consequently, social synchronization may prove to be a bio-behavioral marker of the social deficits in ASD.

In addition, the dissociation of deficits in intentional and spontaneous social synchronization suggests that these kinds of entrainment may function independently and have distinct underlying mechanisms. One might argue that these differences could be due to the fact that the participants with schizophrenia were adults and the participants with ASD were adolescents. This seems unlikely, however, because the data from the adolescent controls replicated the dynamical model predictions that have been extensively demonstrated with adult participants. Another important difference between schizophrenia and ASD is that schizophrenia typically has an onset in early adulthood while the onset of ASD is much earlier and could account for the disruptions in spontaneous synchronization evident in ASD but not schizophrenia. Caution, therefore, is warranted in drawing firm conclusions until future research has explored these differences with larger sample sizes, conducted studies to directly compare diagnostic groups, and compared adult and adolescent populations to isolate any developmental differences.

Our results demonstrating that social synchronization successfully differentiates adolescents with and without ASD is consistent with other work using dynamical measures of synchronization that has found similar differences in social synchronization abilities in children with ASD (ages 6–10 years old; Fitzpatrick et al., 2013a,b). These findings are also consistent with behavioral-coding work indicating disruptions in synchronization of parent–child interactions (Condon, 1982; Trevarthen and Daniel, 2005; Feldman, 2007), timing of facial mimicry (Oberman et al., 2009), synchronization of speech with a partner (Feldstein et al., 1982), and synchronization of speech and gesture (de Marchena and Eigsti, 2010).

The confirmation of social synchronization differences in an older population using a task that allowed direct dynamical modeling, combined with the finding that the synchronization deficit in ASD is different from the deficit seen in schizophrenia, raises the important possibility that social synchronization could be a bio-behavioral marker for ASD. This research also points to the importance of using objective, dynamical, process-oriented measures of social synchronization to be able to fully evaluate the temporal nature of social synchronization. Our research focused on synchronization in the context of a social motor task. Future research is planned to use this dynamical methodology to explore social synchronization in more naturalistic tasks such as the coordination of whole body movements and speech during conversation tasks. Cross recurrence analysis provides another potentially fruitful dynamical methodology for analyzing the temporal and directional characteristics of interpersonal exchanges (e.g., Richardson et al., 2008; Coco and Dale, 2014). Cross recurrence analysis has demonstrated, for example, that mother–infant gaze patterns become more tightly coupled developmentally (Nomikou et al., 2016), infants with ASD are more likely to lag parent–child vocal exchanges while infants without ASD are more likely to lead (Warlaumont et al., 2010), and children with ASD demonstrated less stable and more deterministic social motor coordination (Romero et al., 2016). Questions remain, however, about whether synchronization differences are due to underlying mechanisms that are social, motor, or due to attention or perceptual processing disruptions. Future research is planned to disentangle the role of motor, attention/perception, and social contributions to social synchronization.

One potential limitation of this research is that the participants were performing the task with their parent. While this was chosen to reduce the anxiety that would be inherent in doing the task with a stranger, it may have contributed to the finding that, in both groups the adolescents always led the parent in the coordination. It is possible that there could be something distinct about the interactions between parent and adolescent that would not generalize to interactions of other social pairs. In addition, due to the heritability of ASD (e.g., Zhao et al., 2007; Hallmayer et al., 2011), the parents of the ASD participants could have symptoms on the ASD spectrum that could also contribute to the synchronization displayed by those pairs. Del-Monte et al. (2013) found that this was the case with first-degree relatives of individuals with schizophrenia—they also demonstrated the same overall pattern of synchronization impairments as individuals with schizophrenia. Alternatively, it is possible that parents of children with ASD over-compensate and adjust their behavior more to match their child. If that were the case, it would suggest the synchronization ability of the adolescents might be overestimated here. Green et al. (2010), for example, found that the proportion of synchronous parental communications increased after parents completed a training program that focused on increasing parental response to communication and action routines In future research, we plan to explore this issue by having participants complete the tasks with a stranger.

Another potential limitation lies in the fact that observed synchronization is the result of “live” reciprocal interactions between people. This means that factors of the interaction are by its very nature uncontrolled. This could be circumvented in future research by using video-based presentation of the partner as this would not only allow for the standardization of the movements of the partner used to elicit social behavior, but also for the manipulation of the reciprocity of coupling between the partner and adolescent and the simultaneous recording of the movements of both. This sort of precision in presentation and manipulation of social movements and simultaneous measurement of the user interaction as it unfolds will help clarify the unique contribution of each partner to initiating and maintaining the social synchronization.

Furthermore, the relatively small number of participant pairs used suggests that replication would be prudent before large-scale conclusions can be drawn about the specific pattern of results being a bio-behavioral marker unique to ASD. That being said, it should be noted that our significant effects have large effect sizes and the sample size used here is similar to those in past studies that have investigated social synchronization using the pendulum paradigm with populations without social deficits (Schmidt and O’Brien, 1997; Richardson et al., 2005). Nonetheless, studies including large samples of both ASD and schizophrenia dyads should be performed to definitely conclude that the social synchronization deficits are different for these two groups.

An inherent challenge in delineating the precise nature of ASD-specific social deficits lies in the fact that the population of individuals with ASD is phenotypically and behaviorally heterogeneous. The participants in our sample were relatively high-functioning. In future work we plan to investigate the heterogeneity in adolescents with ASD by recruiting a more diverse participant population and measuring behavior across multiple domains (motor, social, cognitive, emotional, neural) and conducting a discriminant analysis to estimate the contribution each of these components makes to the synchronization difficulties both on the group and individual level. This will allow us to better understand the heterogeneity in ASD and how it relates to synchronization ability.

To help identify the mechanisms underlying intentional and spontaneous synchronization, additional research is planned using electroencephalogram (EEG) and functional magnetic resonance imaging (fMRI) to map brain activity during social synchronization to determine how neurophysiological activity in individuals with ASD is different from that of controls. Researchers are beginning to investigate the underlying neural activity involved in social synchronization (Kelso et al., 2013) but little is known about how it develops or how it may differ between healthy and ASD populations. Research has found that EEG activity in the alpha-mu band between the centroparietal regions in the right hemisphere (Dumas et al., 2010; Naeem et al., 2012) is different during intentional and spontaneous coordination. In particular, those investigators found comparatively more mu suppression in central–parietal brain regions, with intentional synchronization showing more mu suppression than spontaneous. Mu activation is associated with understanding and coordinating motor acts and these patterns of deactivation of mu activity suggest they may be a neural correlate of social synchronization. Exploring whether mu activation is different in ASD during intentional and spontaneous social synchronization could provide us with important insights for understanding the mechanisms responsible for the social problems characteristic of the disorder.

Coordinating one’s movements with another person typically helps to facilitate social connection. The current findings suggest that adolescents with ASD have disruptions in social synchronization and this may in turn interfere with the formation and maintenance of social bonds. The role of abnormal movement patterns during social interactions, and how they may contribute to or maintain social deficits, raises important questions for understanding the social problems characteristic of ASD as well as other developmental and psychiatric disorders. The findings here suggest there may be a social synchronization deficit that is ASD-specific and could likely serve as an objective, bio-behavioral marker.

## Author Contributions

PF: study design, data collection, data analysis, data interpretation, writing; JF: study design, data collection, data analysis, data interpretation, writing; TM: data interpretation, writing; DC: administering clinical assessments, data collection; CC: data recruitment, data collection, data entry; RS: study design, data collection, data analysis, data interpretation, writing.

## Conflict of Interest Statement

The authors declare that the research was conducted in the absence of any commercial or financial relationships that could be construed as a potential conflict of interest.
